# An update in the MRI features, classification, and associated brain abnormalities in hypothalamic hamartomas

**DOI:** 10.1093/bjro/tzag015

**Published:** 2026-06-23

**Authors:** Elham Rahimian, Majid Reza Tahsini, Neda Azin, Soudeh Ghafouri-Fard, Jafar Mehvari Habibabadi, Seyed Sohrab Hashemi Fesharaki, Mohammad Aidin Farahvash, Sara Farahvash, Mahdis Morovvati, Sotirios Bisdas

**Affiliations:** Haghighat Medical Imaging Research Centre, Haghighat Medical Imaging Centre, Tehran 16499, Iran; Haghighat Medical Imaging Research Centre, Haghighat Medical Imaging Centre, Tehran 16499, Iran; Haghighat Medical Imaging Research Centre, Haghighat Medical Imaging Centre, Tehran 16499, Iran; Department of Medical Genetics, Shahid Beheshti University of Medical Sciences, Tehran 19839, Iran; Neuroscience Research Centre, Isfahan University of Medical Sciences, Isfahan 81746, Iran; Pars Advanced & Minimally Invasive Medical Manners Research Centre (PAMIMRC), Tehran 14159, Iran; Roozbeh Hospital, Faculty of Psychiatry, Tehran University of Medical Sciences, Tehran 13185, Iran; Haghighat Medical Imaging Research Centre, Haghighat Medical Imaging Centre, Tehran 16499, Iran; Haghighat Medical Imaging Research Centre, Haghighat Medical Imaging Centre, Tehran 16499, Iran; Department of Translational Neuroscience and Stroke, Queen Square Institute of Neurology, University College London, London WC1N 3BG, United Kingdom

**Keywords:** hypothalamic hamartoma, MRI, magnetic resonance spectroscopy, neurofibromatosis type 1, focal cortical dysplasia

## Abstract

**Objectives:**

Hypothalamic hamartomas (HHs) are rare, non-neoplastic malformations associated with gelastic seizures and, less frequently, precocius puberty. Recent classifications have linked HH subtypes with clinical features, and emerging evidence suggests that HH may co-occur with other brain abnormalities. This study investigates the imaging characteristics of HH, the prevalence of concurrent structural abnormalities, and metabolic alterations using magnetic resonance spectroscopy (MRS)

**Methods:**

We retrospectively analyzed MRI and MRS findings in 18 patients with HH (mean age, 9.24 ± 8 years). Lesions were classified per Li et al, assessing morphology, signal characteristics, associated abnormalities, and MRS-derived choline-to-N-acetylaspartate (Cho/NAA) ratios.

**Results:**

Fourteen patients (77.8%) had gelastic seizures, while 4 had non-seizure-related HH, including precocious puberty (*n* = 1) and NF1 (*n* = 3). HH lesions were isointense on T1-weighted MRI; 88.9% were isointense and 11.1% hyperintense on T2. Concurrent brain abnormalities were found in 50%, including focal cortical dysplasia, amygdala enlargement, and corpus callosum hypoplasia. MRS in 3 cases showed elevated Cho/NAA ratios.

**Conclusion:**

Hypothalamic hamartoma is frequently associated with structural abnormalities and metabolic alterations, particularly in seizure-related cases. These findings support the broader spectrum of HH-related brain abnormalities and highlight the need for further research.

**Advances in knowledge:**

This study provides new insights into the imaging and metabolic characteristics of HH, highlighting the significant association between HH subtypes, brain abnormalities, and metabolic alterations. Additionally, it introduces the elevated choline-to-N-acetylaspartate (Cho/NAA) ratio as a potential biomarker, contributing to a deeper understanding of the underlying pathophysiology and diagnostic approach for HH.

## Introduction

Hypothalamic hamartomas (HHs) are rare, non-neoplastic lesions caused by the abnormal aggregation of neural cells and the formation of disorganized nodules^1–3^ that arise from the hypothalamus, specifically in the region between the mammillary bodies and the infundibulum.^4^ Clinical symptoms of HH, which typically emerge during the first or second decade of life, with a higher prevalence in males compared to females, include gelastic seizures, precocious puberty, or a combination of both. However, the condition can also remain asymptomatic.^5^ On conventional MRI, HH usually appear as round or oval masses with sizes ranging from a few millimeters to several centimeters. They exhibit signal intensity like gray matter on both T1- and T2-weighted sequences, although they may occasionally appear relatively hyperintense on T2-weighted images. Magnetic resonance spectroscopy (MRS) often reveals reduced levels of N-acetylaspartate (NAA) and increased levels of myo-inositol (MI).^1^^,^^6^ Several classification systems have been proposed, based on clinical and imaging features, such as the site of origin, type of attachment, size, and extent of the lesion. Treatment strategies are frequently guided by these classifications.^7–9^ HH may occur as an isolated anomaly or as part of genetic syndromes, such as neurofibromatosis type 1 (NF1) and Pallister-Hall syndrome (PHS).^10^^,^^11^ Comprehensive data on the incidence of associated abnormalities in HH cases are lacking in the literature.^12–14^ Among the possible concurrent conditions to HH brain lesions, the prevalence and clinical significance of cortical developmental abnormalities also remain controversial.

Given the rarity of this entity and the fragmented documentation of their imaging characteristics in relation to their classification subtype and the lack of information about their metabolic makeup, in this study, we present a detailed analysis of 18 classified HH cases with comprehensive analysis of their MRI and MRS profiles, along with evaluation of any coexisting brain abnormalities and their potential relationship to the classified subgroups.

## Methods

Ethical approval was obtained for this study, and patient informed consent was waived due to the study’s retrospective nature.

This was a retrospective study, and diagnostic MRI examinations and clinical information from these cases were obtained from electronic patient records and the neuroimaging repository at a large tertiary neuroimaging referral center. We applied no age limits. All eligible patients were selected based on a clinical history of Gelastic seizures, precocious puberty, or hamartomatous enlargement of the hypothalamus in cases of NF1. For inclusion, the following criteria were used to establish the neuroradiological diagnosis of HH: (1) an isointense lesion relative to normal grey matter on T1-weighted MRI, (2) a sessile or pedunculated lesion connected to the hypothalamus, and (3) the absence of contrast enhancement.^2^^,^^15^^,^^16^ Post-contrast imaging was not performed for all patients; therefore, the last criterion was applied only in cases where post-contrast imaging was available. Exclusion criteria were: (1) prior hypothalamic surgery/ablation that could distort the hamartoma on MRI; (2) unequivocal alternative pathology (eg, enhancing hypothalamic glioma); and (3) non-diagnostic image quality due to motion or incomplete core sequences. The presence of other neurological conditions did not preclude inclusion if the HH criteria were met. Some cases had serial imaging studies, all of which were considered but not included as separate cases.

The imaging was conducted using 2 1.5 T and 1 3 T MR scanners (GE 1.5 T Signa Explorer, 1.5 T Siemens Avanto, and 3.0 T Siemens Trio). Visual inspection was used to assess the characteristics of the lesions, including signal intensity. 3D T1-weighted and T2-weighted sequences were utilized to measure the maximum diameter and signal pattern of the HHs. Signal patterns were compared with the ipsilateral amygdala.

Corpus callosum (CC) morphology was evaluated qualitatively; where thickness/morphology was borderline, interpretation was made relative to **age-appropriate normative CC dimensions** as reported in the pediatric MRI literature.^17^

Additional imaging sequences, including fluid-attenuated inversion recovery (FLAIR), diffusion-weighted imaging (DWI) were also performed.

^1^H magnetic resonance spectroscopy (MRS) was acquired opportunistically in 3 patients based on: lesion dimensions sufficient to accommodate a single voxel with minimal CSF contamination; voxel placement feasibility (eccentric lesions abutting CSF were avoided); scanner time/clinical workflow on the day of MRI; and motion tolerance/sedation availability in younger children. These pragmatic factors, rather than clinical severity, determined which cases underwent spectroscopy. ^1^H MRS was performed using single-voxel spectroscopy (SVS). A TE of 135 ms was selected to enhance spectral resolution by minimizing short-T2 contributions, such as fat and macromolecules, while maximizing the peaks of interest. Post-processing was performed using the vendor’s software, yielding distinct peaks with narrow FWHM for N-acetyl aspartate (NAA) (2.02 ppm), choline (3.2 ppm), and creatine (3.03 ppm) metabolites. Detailed acquisition parameters for all sequences, including ^1^H MRS, are provided in the Supplementary Material.

Hypothalamic hamartomas were classified using the most recent classification system proposed by Li et al.^8^ This system categorizes tumors based on their attachment to the floor of the third ventricle or the hypothalamus. Type I refers to tumors with a narrow interface, while Type II refers to tumors with a broad interface. Tumors are classified as Type III if there is extensive attachment to the third ventricle’s floor extending to the interpeduncular fossa. As Type IV, if there is no extension to the interpeduncular fossa.

Meticulous examination of the remainder of the brain regions to identify additional abnormalities was performed. Possible associated abnormalities included cortical malformations, abnormal gyration patterns, abnormalities in the hippocampus and amygdala (ie, size, shape, and signal intensity), and abnormalities in the appearance of white matter, including the CC, ventricles, basal ganglia, brainstem, cerebellar hemispheres, major vessels, or other developmental anomalies.

Statistical analyses were performed using SPSS software SPSS Science, Chicago, IL, USA and quantitative data were reported as mean ± SD, and qualitative data were reported as frequency distributions (percentages).

## Results

Eighteen patients, 6 females (33.6%) and 12 males (66.7%), were included. The patients’ ages ranged from 16 months to 32 years, with a mean (±SD) age of 9.24 ± 8.00 years. The mean maximum diameter of the HH was 13.72 ± 4.95 mm.

Among the patients, 14 out of 18 (77.8%) presented with gelastic seizures, while 4 out of 18 (22.2%) had non-seizure-related HH, including 1 case of precocious puberty and 3 cases diagnosed with neurofibromatosis type 1 (NF1). All lesions exhibited an isointense signal to the Gray matter on T1-weighted (T1W) sequences. On T2-weighted (T2W) sequences, 16 out of 18 (88.9%) were isointense to Gray matter, while the remaining showed T2W hyperintensity. FLAIR imaging was available for 11patients, all of whom demonstrated increased signal intensity. Contrast-enhanced imaging was performed in 11 patients, none of whom showed enhancement (eligibility criterion). DWI was conducted in 4 patients, and no cases demonstrated diffusion restriction (Table 1).

**Table 1:** Demographics, clinical manifestations, and imaging features in 18 patients with hypothalamic hamartomas.

Feature	**Value** ± **SD (%)**
Gender	Male: 12 (66.7%)/Female: 6 (33.6%)
Age (years, mean ± SD)	9.24 ± 8.09
Maximum diameter (mm, mean ± SD)	13.72 ± 4.95
Gelastic seizure	14/18(77.8%)
Precocious puberty	1/18 (5.5%)
Neurofibromatosis history	3/18(16.7%)
T1W iso-intensity	18/18 (100%)
T2W iso-intensity	16/18(88.9%)
T2W hyperintensity	2/18 (11.1%)
FLAIR hyperintensity	11/11 (100%)
No enhancement on post-contrast T1W	11/11 (100%)
No restriction on DWI	0/4 (100%)

Data available only in patients with the corresponding sequence: FLAIR & post-contrast T1W available in 11/18 patients; DWI available in 4/18 patients.

Regarding associated abnormalities in other brain regions, 3 out of 18 (16.7%) of the patients with gelastic seizures had cortical malformations and abnormal gyration, raising suspicion for focal cortical dysplasia (FCD). Additionally, subependymal heterotopia was identified in 2 out of 18 patients (11.1%).

**Figure 1:**
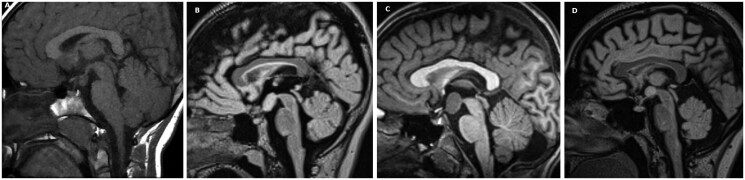
Classification of HH according to Li et al. (8) based on the attachment of the lesion to the hypothalamus and the floor of the third ventricle. (A) Type I: The mass is attached to the hypothalamus or the floor of the third ventricle with a narrow interface. (B) Type II: Same features as Type I, but the mass has a wider interface without causing deformation of the third ventricle floor. (C) Type III: The mass extends superiorly into the third ventricle and inferiorly into the interpeduncular cistern (“straddling” type), deforming the third ventricle. (D) Type IV: The mass is located on the floor of the third ventricle.

Furthermore, among patients with gelastic seizures, 3 out of 18 had hypoplastic CC. An abnormally enlarged amygdala was observed in 3 out of 18 patients (16.7%). Other abnormalities in 3 patients (16.7%) included prominent perivascular spaces in 2 out of 18 patients and a developmental venous anomaly (DVA) in 1 out of 18 patients. No abnormalities were detected in the hippocampus in any of the evaluated cases (Table 2 and Figure 1A).

**Table 2:** Frequency of concurrent brain abnormalities and their classification in patients with gelastic seizures.

Synchronous brain abnormality	Number (%)	Classification—number
Suspicious FCD	3/18 (16.7%)	III—1 IV—2
Hypoplastic CC	3/18 (16.7%)	III—2 IV—1
Amygdala enlargement	3/18 (16.7%)	III—2 IV—1
Prominent perivascular spaces	2/18 (11.1%)	III—1 IV—1
Subependymal heterotopia	2/18 (11.1%)	III—2
Developmental venous anomaly	1/18 (5.5%)	III—1

**Figure 2:**
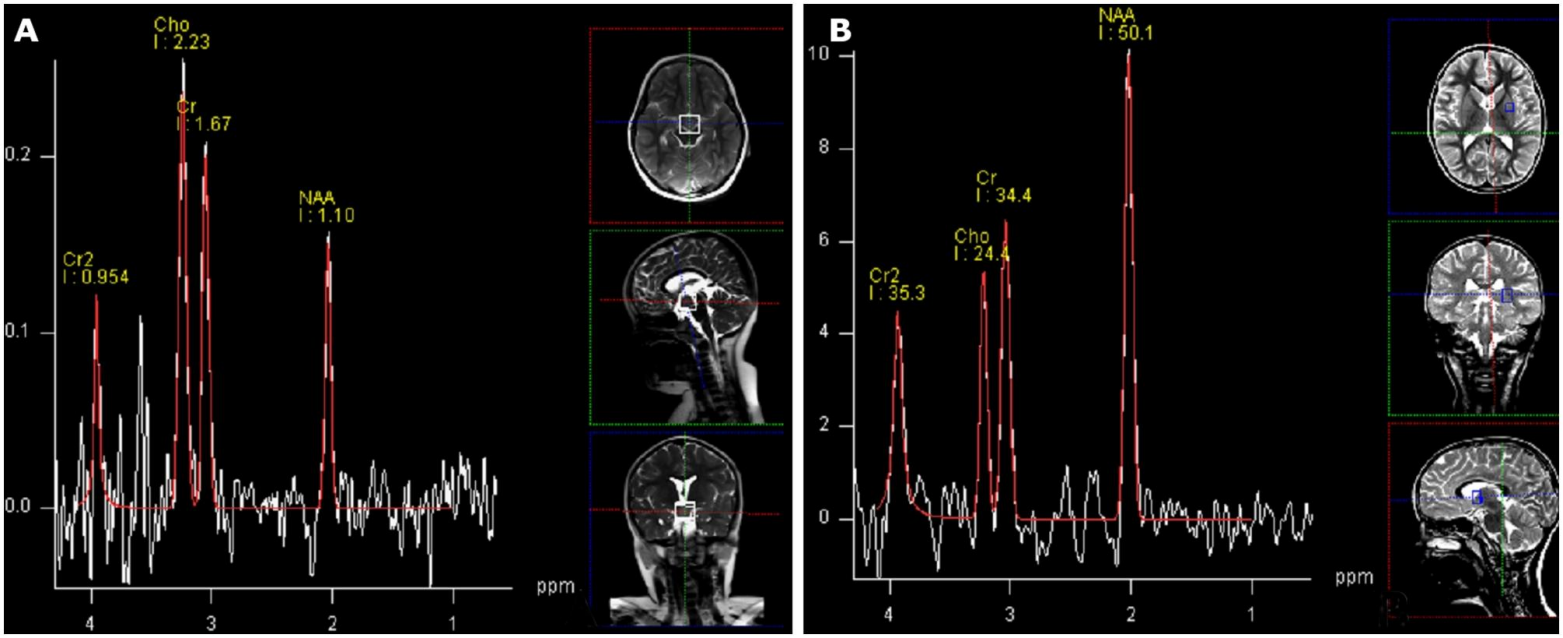
Magnetic resonance spectroscopy findings in hypothalamic hamartoma. Magnetic resonance spectroscopy (MRS) shows a notably higher Cho/NAA ratio in a hypothalamic hamartoma (HH) tumor (A) compared to healthy controls (B).

Magnetic resonance spectroscopy was performed in 3 cases of hamartomatous lesions, and the findings were semi-quantitatively evaluated against an internal database of MRS results in the temporal white matter from age-matched healthy individuals (Table 3). The comparison of Cho/NAA ratios between the 3 patients and their healthy control groups revealed notable differences in all cases, with patients consistently exhibiting a higher Cho/NAA ratio (see Figure 2).

**Table 3:** Comparison of Cho/NAA ratios between patients and healthy controls, including age, mean Cho/NAA ratios, and SD and *P-*value from *t*-test.

Patients number and age (no, in years)	Healthy control group (no, mean age in years)	**Cho/NAA—patient (mean** **±** **SD)**	**Cho/NAA—healthy (mean** **±** **SD)**
(1, 1.5)	Group 1 (4, 1.21)	1.16	0.81 ± 0.12
(2, 3.0)	Group 2 (5, 3.8)	1.35 ± 0.53	0.75 ± 0.14
(1, 7.0)	Group 3 (2, 6.0)	2.03	0.74 ± 0.06

All HH patients with additional significant abnormal findings, such as abnormal gyration and suspected FCD, subependymal heterotopia, and amygdala enlargement, had a history of gelastic seizures and were classified under Types III and IV (Table 2).

## Discussion

**Figure 3:**
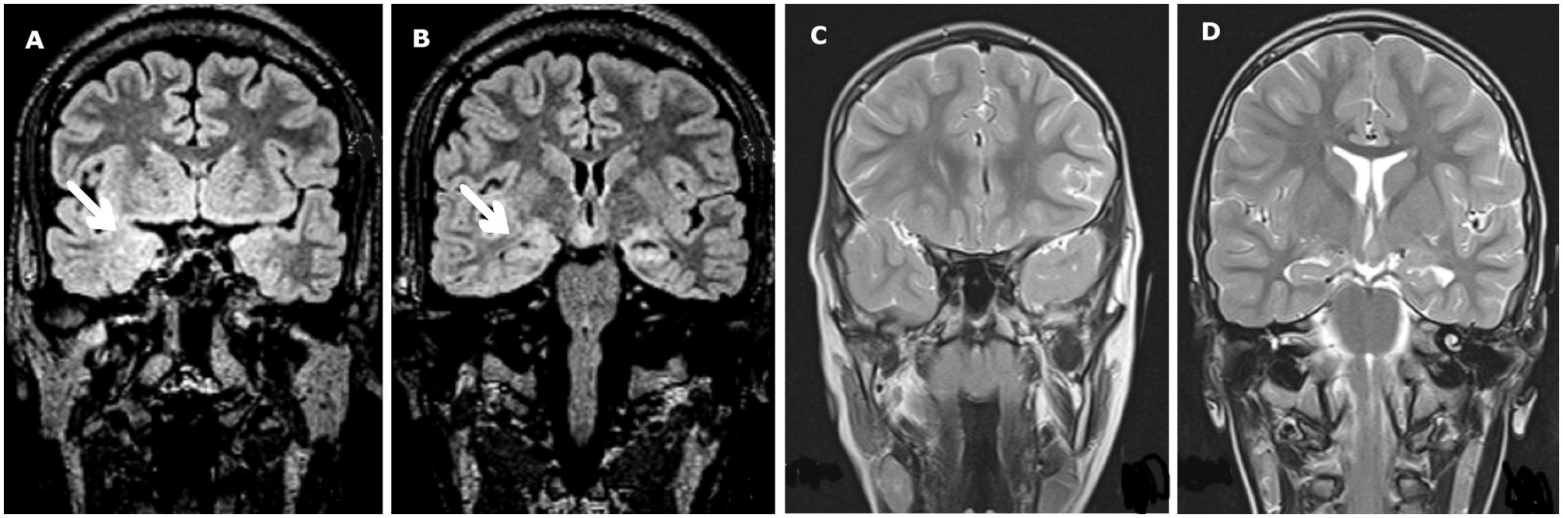
Two HH patients with prominent signal abnormalities suggestive of FCD. (A–B) A patient with blurred grey-white matter junction in the right temporal lobe (arrows) compared to the left. (C–D) A patient with abnormal cortical thickening in the left inferior frontal gyrus, highly suggestive of FCD.

The first anatomical classification system for HH identified 2 groups: the sessile and pedunculated types, based on their attachment patterns to the hypothalamus and the floor of the third ventricle. This classification bears clinical significance because sessile hamartomas are strongly associated with seizures, whereas pedunculated hamartomas are more commonly linked to precocious puberty.^1^^,^^18^ However, the most recent classification, proposed by Li et al and based on 214 cases, was selected in this study. According to this new classification, the most common HH types in our work were Types III and IV.^8^ Given that these types are frequently associated with gelastic seizures, our findings align with previous studies. One of our cases with precocious puberty was classified as Type I, which is also consistent with earlier research, and 1 patient with gelastic seizures was classified as Type II^8^ (Figure 3).

The increased Cho/NAA ratio in HH tumors, compared to the temporal white matter in healthy controls, likely results from elevated choline levels due to increased cell membrane turnover and reduced NAA levels from neuronal dysfunction or rarefaction.^1^^,^^11^^,^^19^ These changes shed light on the tumor’s microenvironment and metabolic activity. The distinct metabolic profile suggests that the Cho/NAA ratio could serve as a biomarker; however, larger studies are needed to confirm these findings and explore the underlying mechanisms.

A significant aspect of the HH imaging spectrum is any co-occurrence of other brain abnormalities. A study by Freeman et al analyzed 72 cases reported to have HH-associated findings. Seventeen patients had associated abnormalities, including anterior temporal white matter signal abnormalities (16%) and arachnoid cysts (6%). However, 2 cases showed subependymal nodular heterotopia and posterior parahippocampal cortical thickening with blurring of the grey-white matter junction, suggestive of FCD.^12^

**Figure 4:**
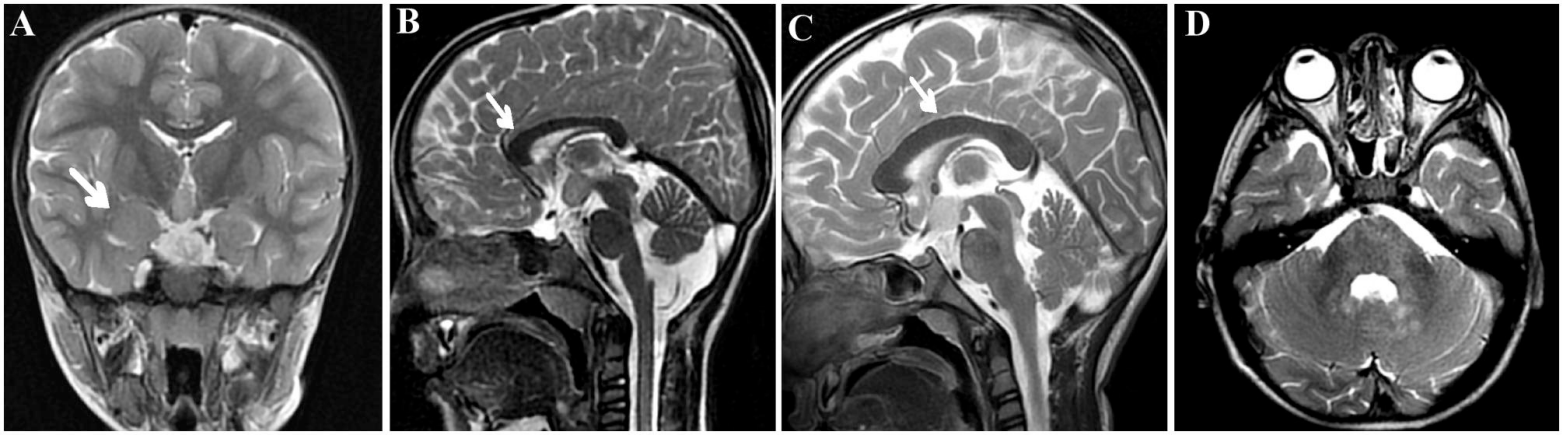
Four HH patients demonstrating various abnormalities in the remaining brain parenchyma. (A) Asymmetrical enlargement of the right amygdala (arrow) in a type IV case. (B) Mild hypoplasia of the corpus callosum (arrow). (C) A case of NF1 with thickened corpus callosum (arrow). (D) Multiple focal areas of signal intensity (FASI) in the remaining brain parenchyma.

Another case report showed an associated HH with subependymal heterotopia and polymicrogyria.^14^ In our work, 4 patients exhibited abnormal gyral patterns, including blurring of the grey-white matter junction in the frontal and temporal lobes, which is highly suggestive of FCD. While the exact link between HH and other brain abnormalities remains unclear, Fenoglio et al suggested that histopathological changes in HH resemble those found in cortical dysplasia and cortical tubers.^20^ This aligns with the hypothesis that HH is a tumor-like dysplastic lesion rather than a true neoplasm^16^ (Figure 4).

To our knowledge, no prior study has systematically evaluated amygdala abnormalities in HH cases. The amygdala has strong anatomical connections with the hypothalamus via the ventral amygdalofugal pathway and the stria terminalis.^21^^,^^22^ In our study, 3 cases with gelastic seizures exhibited amygdala enlargement, with eccentric HH extending toward the side of the amygdala, classified as Type III or IV (Figure 1A). This finding suggests that amygdala involvement should be carefully assessed in all HH cases as a key structure in seizure semiology, particularly regarding fear or anxiety-related symptoms.

A previous case report described an association between HH and CC agenesis, emphasizing its relevance for treatment planning.^13^ The interpretation of CC hypoplasia versus thickening was made in the context of age-referenced norms, which is essential given the developmental variation across childhood.^17^ In the present study, 6 cases demonstrated abnormal callosal configuration, including hypoplasia and thickened CC. All patients with thick CC had underlying NF1, which may be the underlying etiology; however, CC hypoplasia, observed in 3 cases, is likely more closely related to standalone HH (Figure 1B).

**Figure 5:**
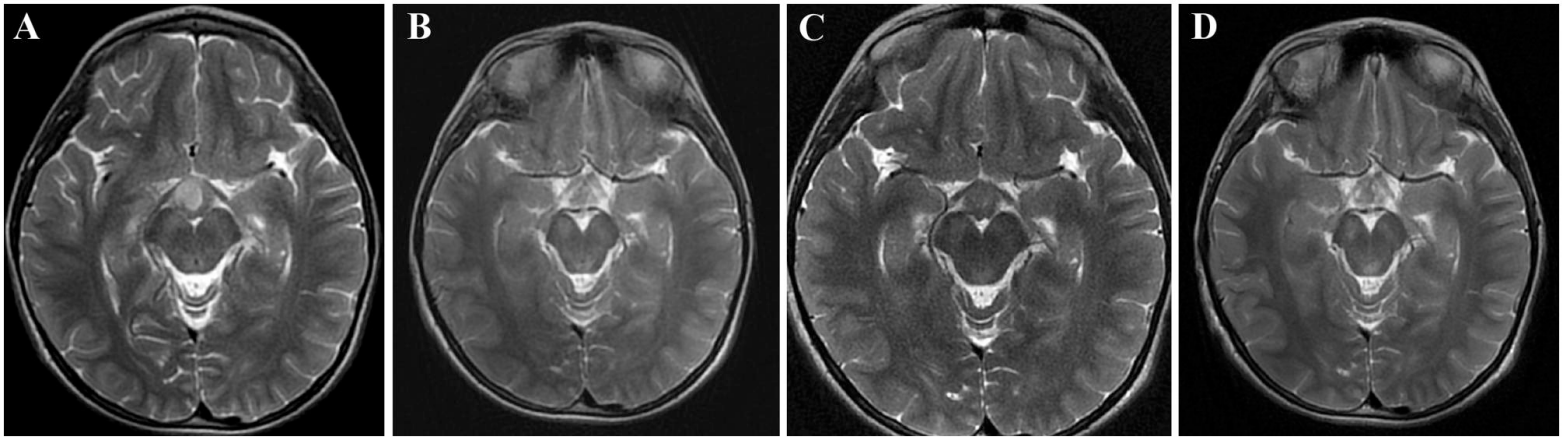
Serial MRI of a patient with NF1-related HH. (A) Initial MRI showing a T2-weighted hyperintense HH in the third ventricular floor. (B) Follow-up MRI after 1 year, demonstrating over 50% reductions in size. (C) MRI taken another year later, showing a slight enlargement of the HH. (D) Subsequent MRI revealing a reduction in HH size once again.

NF1 is a well-known phakomatosis disorder associated with HH. However, in NF1-related HH, precocious puberty and seizures are uncommon, and the lesions typically have a sessile configuration, located in the inferior third ventricle.^6^ In NF1, focal areas of signal intensity (FASI**)** appear as white matter hyperintensities on T2-weighted MRI and exhibit variable behavior. In some patients, these lesions remain stable or even decrease in size with age.^23–25^ In contrast, growing lesions may increase in size or number and demonstrate altered diffusion metrics, such as rising ADC values.^26–28^ We propose that a similar fluctuation in size may also occur in NF1-related HH. In our study, 3 patients with NF1 exhibited a thickened CC and hamartomatous hypothalamic enlargement, classified as type III or IV (Figure 1C and D). In 1 case, 4 follow-up MRIs were performed over a 1-year interval. The second-year MRI objectively showed a 50% reduction in HH size, followed by a 20% enlargement on the third scan. However, the fourth MRI revealed another reduction in size. To the best of our knowledge, this is the first report of such a pattern. These findings suggest that NF1-related HH cases may follow a unique course, potentially requiring a specialized approach (Figure 5).

No patient in our cohort underwent research genetic testing; the genetic and molecular mechanisms discussed below are drawn from prior literature and are included to contextualize HH pathobiology.

Hypothalamic hamartoma is associated with abnormal function of the Sonic hedgehog (Shh) pathway. This pathway plays a role in regulating neurogenesis and cell patterning during the early phase of hypothalamic development. In fact, mutations in the Shh pathway can lead to the upregulated proliferation of nearby wild-type cells.^29^ A combination of various molecular techniques has led to the identification of somatic mutations in Shh pathway-related genes in a proportion of tissue and cell samples from patients with non-syndromic HH.^30^ Another study, involving 9 cases of HH, including 8 non-syndromic cases, has identified pathogenic somatic variants in GLI3, OFD1, and PRKACA in 7 of the 9 cases.^31^ Notably, a 2-hit mutational event, including a germline variant (predicted to disrupt kinase activity) and a somatic loss of heterozygosity, has been detected in TNK2 in a single case of HH. This gene encodes a brain-expressed tyrosine kinase. Thus, TNK2 has been suggested as a novel putative gene contributing to the pathogenesis of HH.^31^

Moreover, germline mutations in *GLI3*, a transcriptional activator and repressor of downstream Shh pathway targets, have been linked to a syndromic form of HH, specifically PHS. Notably, patients with PHS have other clinical manifestations, such as polydactyly or syndactyly, bifid epiglottis, imperforate anus, and renal abnormalities.^32^

Our findings reinforce the role of somatic variants in Shh and cilia genes in HH cases while also shedding light on TNK2 as a potential novel disease-causing gene. This study emphasizes the increasing importance of brain mosaicism in epilepsy disorders and underscores the critical role of genetic diagnosis derived from resected brain tissue.

Several congenital syndromes have also been linked to HH1,^11^ but in our study, none of the cases had an associated syndromic genetic disorder. However, we identified prominent Virchow-Robin spaces in 2 instances and developmental venous anomalies in 1 case, though these findings were considered nonspecific. A previous study suggested a possible relationship between HH and brain cystic abnormalities.^1^ Constitutional mismatch repair deficiency (CMMRD), involving biallelic mutations in mismatch repair genes, is associated with brain findings such as developmental venous anomalies (DVA), FASI, and agenesis of the CC with or without grey matter heterotopia.^33^^,^^34^ Therefore, we postulate that a shared pathway involving DNA repair deficiencies may be responsible for HH and those brain structural aberrations. Furthermore, mutations in the Shh pathway, which regulates hypothalamic development, may contribute to HH formation by promoting the increased proliferation of wild-type cells.^35^ Since pathogenic variants in the Shh signaling pathway and its regulators have been identified in patients with HH,^31^ patients with HH may present with other brain abnormalities due to potentially shared genetic pathways.

Li et al suggested a strong association between type III and IV hamartomas and gelastic seizures.^8^ Freeman et al found that HH patients with gelastic seizures presented with abnormal cortical thickening, blurring of the Gray-white matter junction, subependymal heterotopia, and FCD.^12^ Other case reports have also linked gelastic seizures with CC hypoplasia and polymicrogyria in HH patients.^13^^,^^14^ In our study, all patients with significant associated abnormalities, such as FCD, amygdala enlargement, CC hypoplasia, and subependymal nodular heterotopia, had Type III or IV HH. This finding further supports an association between more severe types of HH and the presence of variable structural abnormalities with epileptogenic potential. Nonetheless, we acknowledge the main limitation of our study, which is the small sample size for this rare condition and the lack of genetic research in all patients. However, we believe that our work contributes to the growing evidence of an increased prevalence of concurrent structural abnormalities in HH patients.

## Conclusion

The findings in this study provide an update and contribute to previous evidence suggesting that HH may not be viewed as an isolated entity, but rather may be associated with additional brain abnormalities, as described above. Further studies with larger sample sizes of this rare disease are needed to explore these relationships in greater detail, and genetic studies may provide additional insight into the genetic and pathophysiological substrates of HH.

## Supplementary Material

tzag015_Supplementary_Data
