# Supplementary material for: An update in the MRI features, classification, and associated brain abnormalities in hypothalamic hamartomas
Source: BJR Open. 2026 Jun 23;8(1):tzag015. doi: 10.1093/bjro/tzag015 (PMC13342888; doi:10.1093/bjro/tzag015)
Supplement: tzag015_Supplementary_Data [file tzag015_supplementary_data.docx]

Supplementary Material

# MRI and ¹H-MRS Acquisition Parameters

Magnetic resonance imaging was performed using two 1.5T systems (GE Signa Explorer; Siemens Avanto) and one 3T system (Siemens Trio).

The following acquisition parameters were used:

3D T1-weighted MPRAGE:
TR/TE = 2300/3.2 ms;
Field of view (FOV) = 256 × 256 mm²;
Voxel size = 1.0 × 1.0 × 1.0 mm³.

T2-weighted axial imaging:
TR/TE = 4000/106 ms;
FOV = 230 × 230 mm²;
Matrix = 256 × 256;
Slice thickness = 5 mm.

Fluid-Attenuated Inversion Recovery (FLAIR):
TR/TE/TI = 8000/100/2370 ms;
FOV = 230 × 230 mm²;
Matrix = 256 × 256;
Slice thickness = 5 mm.

¹H Magnetic Resonance Spectroscopy (MRS):
Single-voxel spectroscopy (SVS) was performed with TE = 135 ms.
Spectra were acquired and processed using vendor-provided software, yielding peaks for:
- N-acetylaspartate (NAA) at 2.02 ppm
- Choline (Cho) at 3.2 ppm
- Creatine (Cr) at 3.03 ppm.
